# Effects of Three Types of Exercise Interventions on Healthy Old Adults’ Gait Speed: A Systematic Review and Meta-Analysis

**DOI:** 10.1007/s40279-015-0371-2

**Published:** 2015-08-19

**Authors:** Tibor Hortobágyi, Melanie Lesinski, Martijn Gäbler, Jessie M. VanSwearingen, Davide Malatesta, Urs Granacher

**Affiliations:** Center for Human Movement Sciences, University Medical Center Groningen, University of Groningen, A. Deusinglaan 1, 9700 AD Groningen, The Netherlands; Faculty of Health and Life Sciences, Northumbria University, Newcastle Upon Tyne, UK; Division of Training and Movement Sciences, University of Potsdam, Potsdam, Germany; Department of Physical Therapy, School of Health and Rehabilitation Sciences, University of Pittsburgh, Pittsburgh, PA USA; Institute of Sport Sciences University of Lausanne (ISSUL), University of Lausanne, Lausanne, Switzerland; Department of Physiology, Faculty of Biology and Medicine, University of Lausanne, Lausanne, Switzerland

## Abstract

**Background:**

Habitual walking speed predicts many clinical conditions later in life, but it declines with age. However, which particular exercise intervention can minimize the age-related gait speed loss is unclear.

**Purpose:**

Our objective was to determine the effects of strength, power, coordination, and multimodal exercise training on healthy old adults’ habitual and fast gait speed.

**Methods:**

We performed a computerized systematic literature search in PubMed and Web of Knowledge from January 1984 up to December 2014. Search terms included ‘Resistance training’, ‘power training’, ‘coordination training’, ‘multimodal training’, and ‘gait speed (outcome term). Inclusion criteria were articles available in full text, publication period over past 30 years, human species, journal articles, clinical trials, randomized controlled trials, English as publication language, and subject age ≥65 years. The methodological quality of all eligible intervention studies was assessed using the Physiotherapy Evidence Database (PEDro) scale. We computed weighted average standardized mean differences of the intervention-induced adaptations in gait speed using a random-effects model and tested for overall and individual intervention effects relative to no-exercise controls.

**Results:**

A total of 42 studies (mean PEDro score of 5.0 ± 1.2) were included in the analyses (2495 healthy old adults; age 74.2 years [64.4–82.7]; body mass 69.9 ± 4.9 kg, height 1.64 ± 0.05 m, body mass index 26.4 ± 1.9 kg/m^2^, and gait speed 1.22 ± 0.18 m/s). The search identified only one power training study, therefore the subsequent analyses focused only on the effects of resistance, coordination, and multimodal training on gait speed. The three types of intervention improved gait speed in the three experimental groups combined (*n* = 1297) by 0.10 m/s (±0.12) or 8.4 % (±9.7), with a large effect size (ES) of 0.84. Resistance (24 studies; *n* = 613; 0.11 m/s; 9.3 %; ES: 0.84), coordination (eight studies, *n* = 198; 0.09 m/s; 7.6 %; ES: 0.76), and multimodal training (19 studies; *n* = 486; 0.09 m/s; 8.4 %, ES: 0.86) increased gait speed statistically and similarly.

**Conclusions:**

Commonly used exercise interventions can functionally and clinically increase habitual and fast gait speed and help slow the loss of gait speed or delay its onset.

**Electronic supplementary material:**

The online version of this article (doi:10.1007/s40279-015-0371-2) contains supplementary material, which is available to authorized users.

## Key Points

**Table:** 

The aim of this systematic review and meta-analysis was to determine whether therapeutic exercise interventions (resistance, coordination, and multimodal training) have an overall effect on healthy old adults’ gait speed.
Commonly used exercise interventions can substantially but similarly increase healthy old adults’ habitual and fast gait speed.
Healthy old adults and care providers can select among these exercise programs freely and customize each program based on individual preferences, experience, social context, and medical precaution.

## Introduction

Bipedal locomotion is a hallmark of human evolution, and gait speed affords evolutionary [1], medical [2–5], cognitive [6, 7], and health-related [8, 9] benefits to humans across the lifespan, especially to the aged [10–29]. Even healthy aging is associated with evolving muscular, neuronal, and cognitive dysfunctions [30–36], resulting in functional impairments, one of which is a characteristic and clearly recognizable slowing of habitual walking speed by as much as 16 % per decade starting at the age of 60 years [10, 12–14, 21, 25, 37]. Habitual walking speed measured on a level surface predicts many conditions later in life, including daily function [38, 39], mobility [40, 41], independence [42], falls [19, 43, 44], fear of falls [45], fractures [43], health [46], mental health [47], cognitive function [48–51], post-acute transition to the community [52], adverse clinical events [53], hospitalization [38], institutionalization [42], mortality [53–55], and survival [56, 57] (for a review, see Abellan van Kan et al. [10]).

When a 65-year-old senior walks at a habitual gait speed of a 25 year old, this maintained gait speed of 1.2 m/s signifies multi-systemic wellbeing, whereas habitual gait speed below 1.0 m/s at an age over 65 years suggests the presence of potentially clinical or sub-clinical impairments [10]. A reduction of as small as 0.1 m/s in habitual gait speed is associated with a 10 % decrease in the ability to perform instrumental activities of daily living [58]. Recognizing the medical, clinical, physiological, cognitive, and health-related importance of maintaining gait speed in old age, some researchers consider habitual gait speed as the sixth vital sign [59]. A strong consensus is emerging that family physicians should incorporate walking speed in clinical practice as a standard measurement of old adults’ daily function and mobility [4, 60].

Prevention of gait speed loss while being relatively healthy during late mid-life and especially over the age of 65 years is thus a priority. Evidence is overwhelming that high levels of spontaneous physical activity and a variety of forms of systematic exercise can slow the decline of muscular, tendinous, skeletal, nervous, and cognitive function as well as that of other organs, and the correlated physiological benefits can in turn slow the deterioration of activities of daily living, including gait speed [12, 28, 35, 61]. Previous reviews have examined several important concepts related to gait speed, including habitual gait speed as an index of aging [11], the effects of age on gait speed across the lifespan [21], age norms of habitual and fast walking speed [14, 24, 25], the standardization of gait speed testing or a lack of it in clinical settings [2], and how gait speed should be a part of a comprehensive geriatric assessment [4].

Among healthy older adults, much less is known about how specific exercise interventions improve gait speed [12]. A few reviews have examined the effects of physical activity and systematic exercise on gait speed, but conclusions were limited due to a qualitative approach [62], a reliance on a handful of exercise studies selected without specific justification [18], and by the inclusion of old adults with and without comorbidities [2, 63]. A critical issue that has been consistently overlooked in the literature is the comparative efficacy of specific types of exercise interventions on habitual and fast gait speed in healthy old adults. In this context, a particularly relevant review quantified the effects of strength and multimodal exercise interventions on gait speed and found that such therapeutic exercises can improve gait speed in community-dwelling old adults in a dose- and intensity-dependent manner but to such a small extent (0.01 m/s, *p* < 0.05) that therapeutic effects are questionable [64]. Another comprehensive review compared single with multimodal interventions on gait speed and concluded without statistical quantification that “…there is little empirical support that supplementing strength training with other modes of training (such as aerobic, balance and coordination activities) results in further improvement in locomotor function” [8].

To the best of our knowledge, no systematic review and meta-analysis has currently directly specified the combined and individual effects of the most widely used exercise interventions on the habitual and fast gait speed of healthy old adults. Intervention modalities most likely to improve gait speed can be grouped as those targeting impairments, i.e., muscle strength and power [8, 41, 64–68], and as those targeting the timing and coordination elements of gait [16, 28, 69]. Therefore, the primary goal of the present review is to determine the effects of strength, power, coordination, and multimodal exercise training on the habitual and fast gait speed of healthy old adults. Based on the available reviews, the overall hypothesis is that (1) the four intervention types can improve the gait speed of healthy old adults and, perhaps somewhat provocatively, we also hypothesize that (2) these training effects are comparable. Although even healthy old compared with young adults present with substantial reductions in muscle strength [70], muscle power [71, 72], muscle mass [73], incomplete muscle activation [66], sensory dysfunction [74], balance problems [33], coordination deficits [16], and sub-clinical cognitive [48] and mobility impairments, i.e., slow gait [12], we argue that these dysfunctions are evenly and randomly distributed among healthy old adults. Therefore, in the absence of one specific dysfunction among healthy old adults, the adaptations to the four interventions are also heterogeneously distributed, making it unlikely that any one particular or even a multimodal exercise intervention would be superior in increasing gait speed. Some experimental evidence supports this hypothesis based on the similar changes in functional outcomes reported by studies that compared two types of exercise interventions [75–77], but this is not always the case [78]. Further, the often promoted higher efficacy of multimodal versus single-arm interventions can be undermined and any extra effect negated by the potentially unfavorable interaction between individual elements that form a multimodal intervention [79]. Therefore, we determined the effects of resistance, coordination, and multimodal exercise and then we inferred from these data the relative efficacy of each exercise intervention.

Data are also lacking in the gait reviews published so far concerning critical aspects of the gait speed tests. Previous reviews did not categorize or used only a narrow range of distance walked during the gait speed tests (<15 m) [4]. While the patterns of change in 20-m and 20-min walks were similar over an observation period of 8 years [80], it remains unclear and unexplored whether therapeutic exercise interventions would have a homogenous effect on gait speed measured over a short and long distance, each indexing different physiological mechanisms [81]. Currently, information is insufficient for a concept-based hypothesis concerning distance walked during the gait test (short vs. long). Finally, it is equally unclear from the existing literature whether exercise interventions would have a differential effect on gait performance tested at a habitual and fast (‘maximal’) pace. One review, based on limited data, reported zero intervention effects on the fast gait speed of old adults [64], contradicting results of several studies, reporting that strength and endurance training significantly increased the fast gait speed of healthy old adults [76, 82–84]. Because fast compared with habitual walking requires greater limb accelerations produced by muscle forces, our tentative hypothesis is that interventions would be more effective in improving the fast gait speed than the habitual gait speed of healthy old adults. Taken, together, the second aim of the review was to determine the effects of strength, power, coordination, and multimodal exercise interventions on gait speed measured over a short versus a long distance and at a habitual and fast pace. As a forewarning, we state that the search identified only one power training study, therefore the subsequent analyses focused only on the effects of resistance, coordination, and multimodal training on gait speed.

## Methods

### Literature Search and Selection Criteria

We performed a computerized systematic literature search in PubMed, Web of Knowledge, and Cochrane databases from January 1984 up to December 2014. Appendix S1 in the electronic supplementary material (ESM) shows the Boolean search syntax used in PubMed. The PubMed syntax consisted of three main terms and was designed to determine the effects of four types of exercise interventions on the gait speed of healthy old adults. Term 1 focused on four interventions: (1) resistance training, (2) power training, (3) coordination training, and (4) multimodal training and search term variants within each category. Term 2 was the outcome term, focusing on gait speed and its variants. Term 3 was the exclusion term. We also applied the following filters to delimit the search to articles available in full text, publication period over past 30 years, human species, journal articles, clinical trials, randomized controlled trials, English as publication language, and age ≥65 years. We determined the age criterion by averaging the age of subjects across intervention and control groups in a given study and, if this averaged value equaled or exceeded 65, the study was included. The PubMed syntax was then adapted to the search in the Web of Knowledge and Cochrane databases.

We scanned each article’s reference lists in an effort to identify additional suitable studies for inclusion in the database, including reviews [8, 12, 64]. In addition, relevant journals within the sections gerontology/geriatric medicine (e.g., *Age and Ageing*, *Gerontology*, *Journal of the American Geriatrics Society*, *Journals of Gerontology*) were searched for the terms ‘training’ OR ‘intervention’ AND ‘gait speed’ OR ‘walking speed’. Duplicates between searches were removed. We also applied additional filters to exclude studies that were published in non-peer reviewed journals; failed to use at least one measure of gait speed; failed to report the pre–post means and standard deviations numerically or in a graphic form; were case reports; or failed to report or administer minimum requirements regarding training design such as exercise volume, frequency, and intensity. We also note the application of a unique filter, gait speed, our main outcome variable. Because this review targets healthy old adults, we set a minimum pre-intervention gait speed, as recommended for this population in the literature, at 1.0 m/s [10, 59, 85] but lower than 1.0 m/s for tests that included postural tasks and walking on a curved path (i.e., timed-up-and-go test [TUG]) [86]. We also excluded studies that used an active control group. Three independent reviewers (ML, MG, UG) screened citations of potentially relevant publications based on the inclusion and exclusion criteria. If the citation showed potential relevance, it was screened at the abstract level. When abstracts indicated potential inclusion, full-text articles were reviewed for inclusion. A consensus meeting was held with TH if the three reviewers were not able to reach agreement upon inclusion of an article.

### Coding of Studies

Each study was coded for the following variables: age, sex, body mass, height, and number of participants; number and type of interventions; number and type of control groups; walking distance, path (i.e., straight, curved), or duration of gait speed measurement, speed of gait test (fast vs. habitual), and baseline and post-intervention values of gait speed. We also extracted the characteristics of exercise interventions (duration, intensity, etc.) to ascertain the appropriateness of a study for inclusion, but these parameters are not analyzed in the present review. In several cases, we contacted the authors to provide the necessary gait speed data or other pertinent details, but the analyses contain only a few data points estimated from the published figures.

We defined resistance training as a systematic series of exercises that cause muscles to work or hold against an applied force or weight [87] in an effort to increase the ability to produce maximal voluntary force. In contrast, we included interventions under the umbrella term ‘coordination’ that emphasized the use of one’s own bodyweight and had subjects perform balance, walking, dance, functional training, and running in the form of endurance training [16, 76, 88]. For example, functional training was designed to ‘improve daily tasks in the domains first affected in older adults, namely, moving with a vertical component, moving with a horizontal component, carrying an object, and changing between lying-sitting-standing position’ [89]. A resistance and coordination intervention each included only one main type of exercise program. Finally, multimodal interventions were those that included at least two or more types of exercise programs in any combination between resistance, aerobic training, balance, and functional training.

### Assessment of Methodological Quality and Statistical Analyses

The methodological quality of all eligible intervention studies was assessed using the Physiotherapy Evidence Database (PEDro) scale. The PEDro Scale is used to rate internal study validity and the presence of statistical replicable information on a scale from 0 to 10 with ≥6 representing a cut-off score for high-quality studies [90].

To determine the effectiveness of an exercise intervention in relation to gait speed, we computed between-subject effect size (ES) using the implemented formula in Review Manager version 5.3 (Hedges’ adjusted g) as (ES = ±[(mean post-value intervention group) − (mean post-value control group)]/pooled variance) [91]. ESs were calculated only for those comparisons that involved an experimental group and what we refer to as a ‘passive’ or ‘inactive’ control group and was adjusted for respective sample sizes. We used such control groups so that we could consistently determine the effects of an exercise intervention relative to a non-exercise control instead of another intervention group. In addition, weighting of the studies was applied in Review Manager version 5.3 according to the magnitude of the respective standard error. We used the random-effect meta-analysis model in Review Manager to compute overall ESs [92]. Increases in gait speed are reported as a positive change, and such changes are referenced to non-intervention controls so that a positive change in gait speed represents the superiority of an intervention compared with control. The calculation of ES makes it possible to conduct a systematic and quantitative evaluation whether or not exercise interventions versus control interventions affect gait speed and, if so, whether these differences are also of practical importance. ES values of 0.00 ≤ 0.49 indicate small, 0.50 ≤ 0.79 indicate medium, and ≥0.80 indicate large practical effects [93].

## Results

### Study Characteristics

**Fig. 1:**
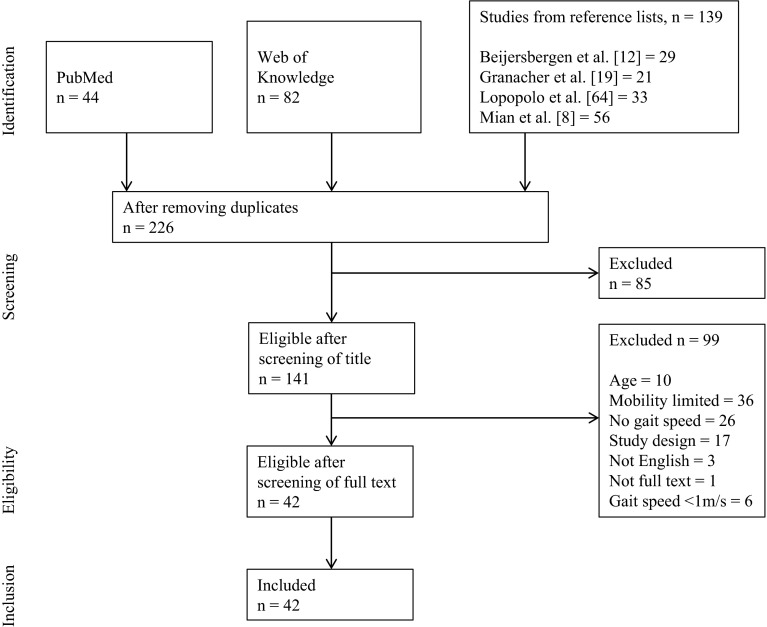
Flowchart illustrating the different phases of the search and study selection

Figure 1 shows the study selection flow chart. The search identified 42 eligible studies from an original search yield of 226 studies [22, 76, 83, 89, 94–131]. Our original intention was to determine the effects of four types of exercise interventions on gait speed. However, the search identified only one study concerning the effects of leg power training on gait speed and we incorporated this study in the resistance training intervention [109].

Table 1 shows the characteristics of the 42 studies included in the analyses. The current analysis is based on 2495 healthy old adults aged 74.2 years (64.4–82.7; the 64.4 value represents the mean age of a control group in one study reference [114]). Because several studies reported only the total number of subjects, the 796 males and 1348 females are only crude estimates of the sex distribution. Body mass (69.9 ± 4.9 kg), height (1.64 ± 0.05 m), body mass index [BMI] (26.4 ± 1.9 kg/m^2^), and gait speed (1.22 ± 0.18 m/s) all suggest that the conclusions of the review are relevant to healthy old adults. Appendix S2 in the ESM shows that the quality of the included studies was low, with a mean PEDro score of 5.0 (±1.2) [90].

**Table 1:** Effects of three types of exercise interventions on gait speed in healthy old adults

Intervention and group	Age, years	*N* (M/F)^a^	BMI, kg/m^2^	*d*, m	Pre, m/s	Post, m/s	∆, m/s	∆, %	ES
Resistance training									
Exp	71.9 (4.2)	613 (216/290)	26.8 (2.1)	12.0 (22.5)	1.22 (0.36)	1.33 (0.43)	0.11 (0.15)	9.3 (10.1)	0.84
Con	72.6 (4.3)	533 (205/257)	26.3 (1.5)	12.0 (22.5)	1.18 (0.16)	1.18 (0.15)	0.00 (0.06)	–0.3 (4.7)	NA
Coordination training									
Exp	74.9 (3.0)	198 (71/97)	26.5 (2.1)	8.7 (1.4)	1.22 (0.18)	1.31 (0.21)	0.09 (0.06)	7.6 (6.5)	0.76
Con	74.9 (4.0)	187 (61/100)	27.9 (1.5)	8.7 (1.4)	1.21 (0.15)	1.19 (0.17)	–0.02 (0.10)	–2.2 (8.8)	NA
Multi-modal training									
Exp	75.6 (4.0)	486 (134/308)	25.2 (2.0)	13.6 (9.5)	1.26 (0.20)	1.35 (0.19)	0.09 (0.16)	8.4 (12.4)	0.86
Con	75.1 (4.5)	478 (109/296)	25.4 (1.9)	13.6 (9.5)	1.21 (0.20)	1.22 (0.19)	0.01 (0.04)	0.9 (3.6)	NA
All									
Exp	74.1 (3.7)	1297 (421/695)	26.2 (2.1)	11.4 (11.2)	1.23 (0.18)	1.33 (0.19)	0.10 (0.12)	8.4 (9.7)	0.84
Con	74.2 (4.3)	1198 (375/653)	26.6 (1.6)	11.4 (11.2)	1.20 (0.18)	1.19 (0.17)	–0.01 (0.07)	–0.6 (5.7)	NA

Values other than frequencies and ES are mean (±SD)

*Con* control, *d* distance used to measure gait speed, *ES* between-group ES (ES ≥0.80 is large), *Exp* experimental, *F* female, *M* male, *Post, m/s* gait speed after intervention, *Pre, m/s* gait speed before intervention, *∆, m/s* change in gait speed, *∆, %* change in gait speed, *NA* not applicable because ES is computed between and not within groups, *SD* standard deviation

^a^The number of females and males are only crude estimates of the sex distribution because many studies reported only a total sample size. Therefore, the values for males and females do not sum to the total sample size, denoted by *N*, used in the analysis

### Primary Analysis: Overall Effects of Three Types of Intervention on Gait Speed

In order to pool studies and to calculate a pooled ES for the primary analyses, we prioritized the inclusion of gait tests being administered over (1) a short and straight distance, (2) long distance, and (3) TUG. If a study reported two gait tests with respect to speed, we prioritized habitual over fast gait speed.

**Fig. 2:**
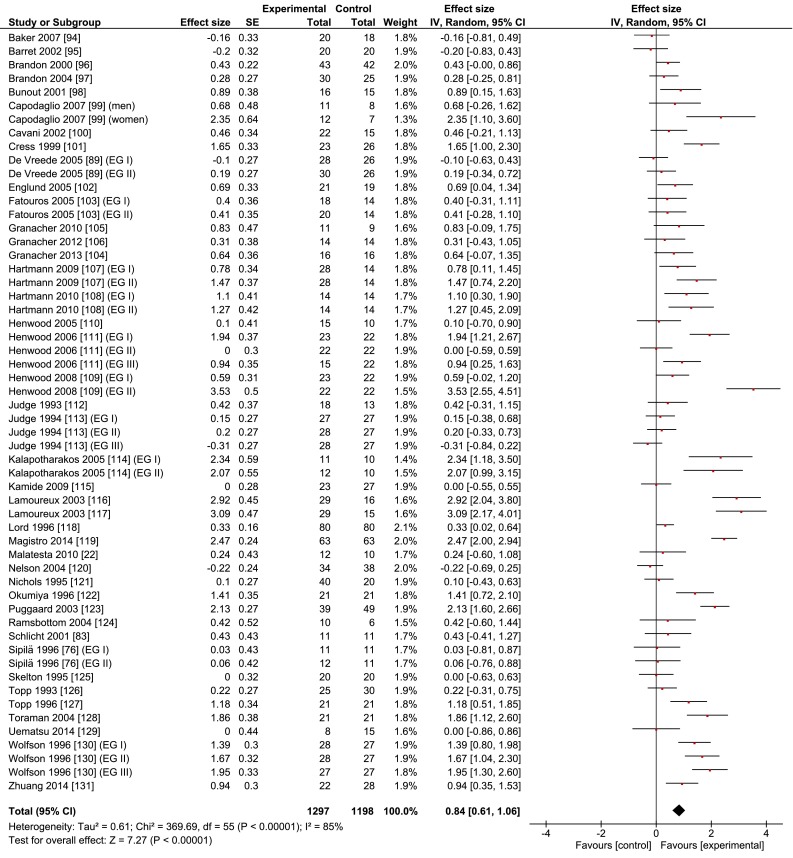
Meta-analysis of the overall effects of resistance, coordination, and multimodal training on the gait speed of healthy old adults. *CI* confidence interval, *IV* inverse variance, *SE* standard error

Table 1 and Fig. 2 show that the three types of intervention improved gait speed in the three experimental groups combined (*n* = 1297) by 0.10 m/s (±0.12) or 8.4 % (±9.7), with a large ES of 0.84. These changes in gait speed were observed in gait tests administered over a variety of distances, with a mean of 19.7 m (±42.1, range 2–471), over straight, curved paths, or an otherwise unspecified path, and at habitual or ‘fast’ walking speed. The ESs ranged from −0.31 to 3.53.

**Fig. 3:**
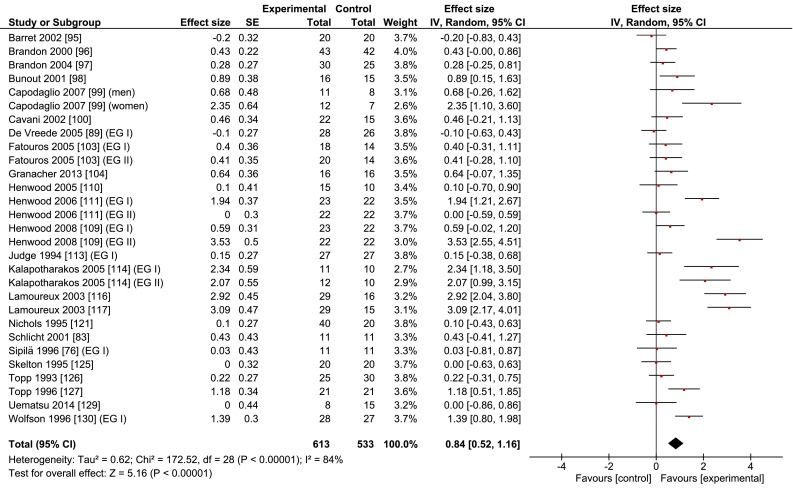
Meta-analysis of the effects of resistance training on the gait speed of healthy old adults. *CI* confidence interval, *IV* inverse variance, *SE* standard error

Table 1 and Fig. 3 show that resistance training (24 studies, *n* = 613) improved gait speed by 0.11 m/s (±0.15, range –0.20 to 0.52) or 9.3 % (±10.1, range −14 to 33) compared with inactive controls (*n* = 533), with a large ES of 0.84 (range –0.20 to 3.53). On average, the resistance training programs lasted 14.6 weeks (±6.6, range 6–26), consisted of 39 sessions (±20, range 30–60), and were delivered at a low to high exercise intensity, quantified as 50–80 % of the one repetition maximum of various leg exercises. The control groups, as defined in this review and by the authors of the included studies, were inactive and ‘maintained normal activity’, but in one study the control group did engage in stretching and light physical activity [95] or received educational information on physical activity [130].

**Fig. 4:**
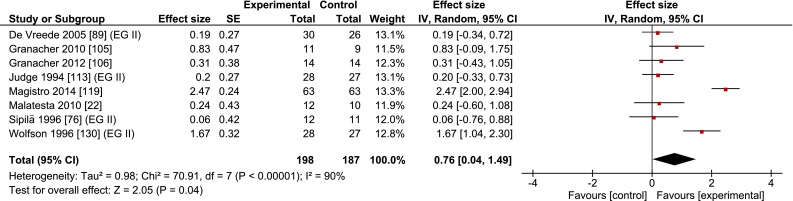
Meta-analysis of the effects of coordination training on the gait speed of healthy old adults. *CI* confidence interval, *IV* inverse variance, *SE* standard error

Table 1 and Fig. 4 show that coordination training (eight studies, *n* = 198) improved gait speed by 0.09 m/s (±0.06, range 0.02–0.15) or 7.6 % (±6.5, range 1.5–19.6) compared with inactive controls (*n* = 187), with a medium ES of 0.76 (range 0.06–2.47). On average, the coordination training programs lasted 11.5 weeks (±4.3, range 6–18) and consisted of 31 sessions (±14, range 16–54). The intensity of such programs is difficult to quantify [132, 133]. The control groups were inactive or in a few cases received educational information about physical activity [76, 130].

**Fig. 5:**
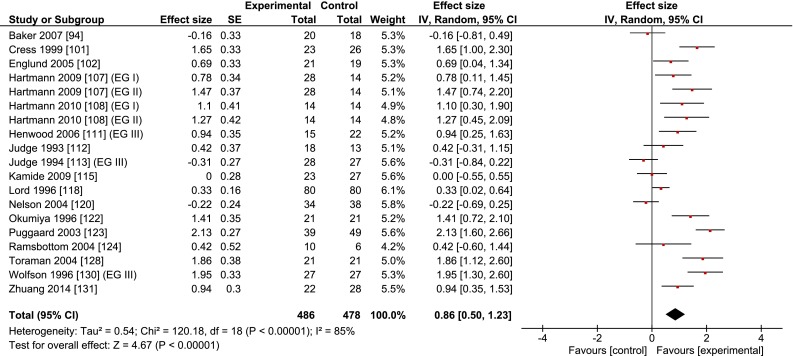
Meta-analysis of the effects of multimodal training on the gait speed of healthy old adults. *CI* confidence interval, *IV* inverse variance, *SE* standard error

Table 1 and Fig. 5 show that multimodal training (19 studies, *n* = 486) improved gait speed by 0.09 m/s (±0.16, range –0.20 to 0.58) or 8.4 % (±12.4, range −12 to 44) compared with inactive controls (*n* = 478), with a large ES of 0.86 (range –0.31 to 2.13). On average, the multimodal training programs lasted 17.7 weeks (±10.2, range 8–47) and consisted of 41.4 sessions (±22.7, range 16–94). The intensity of these programs was characterized as ‘moderate’ [94, 115], ‘hard, very hard’ [107], ‘to volitional fatigue’ [112], or ‘using body weight’ [131]. The control groups were inactive or received educational information about physical activity [120].

### Secondary Analyses: Effects of the Three Types of Interventions on Gait Speed with Respect to the Speed and Distance of Gait Tests

One of the three secondary analyses examined the potential differential effects of the three interventions on gait speed with respect to the speed of the gait test (habitual vs. fast). In the second analysis, we examined the overall effects of the three interventions on gait speed with respect to the distance of the gait test (short vs. long). The third analysis examined the overall effects of the three interventions on the TUG. As in the primary analyses, in order to pool studies and to calculate a pooled ES in these secondary analyses, we prioritized the inclusion of gait tests being administered over (1) a short and straight distance, (2) long distance, and (3) TUG. If a study reported two gait tests with respect to speed, we prioritized habitual over fast gait speed.

Table 2 summarizes the effects of the three types of exercise interventions according to the speed of the gait tests, i.e., habitual versus fast. In 24 of 27 studies, habitual gait speed was tested over a straight path with an average distance of 12.4 m. In 15 of 24 studies, fast gait speed was tested over a straight path with an average distance of 9.3 m. Overall, the three interventions seemed to improve fast gait speed somewhat more (increase of 0.12 m/s, 9.4 %, ES: 0.89, *n* = 750) than habitual gait speed (increase of 0.07 m/s or 5.8 %, ES: 0.94, *n* = 843). Of the three interventions, resistance and coordination training improved habitual gait speed similarly (0.09 vs. 0.08 m/s or 6.8 vs. 6.3 %), with resistance training having nearly twice the ES (1.15 vs. 0.66). Multimodal training had an ES of 0.77 (change of 0.05 m/s and 4.4 %). All three interventions improved fast gait speed numerically identically by 0.12 m/s. Resistance, coordination, and multimodal training improved fast gait speed by 9.0 % (ES: 0.90), 8.7 % (0.73), and 10.5 % (0.94), respectively.

**Table 2:** Effects of three types of exercise interventions on habitual and fast gait speed in healthy old adults

Intervention and group	Speed	*N*	*d*, m	Pre, m/s	Post, m/s	∆, m/s	∆, %	ES
Resistance training								
Exp	Habitual	410	13.9 (27.1)	1.28 (0.25)	1.37 (0.33)	0.09 (0.16)	6.8 (11.2)	1.15
	Fast	368	8.4 (3.4)	1.49 (0.44)	1.62 (0.46)	0.12 (0.08)	9.0 (6.9)	0.90
Con	Habitual	355	13.9 (27.1)	1.20 (0.24)	1.19 (0.23)	–0.01 (0.06)	–0.7 (5.2)	NA
	Fast	320	8.4 (3.4)	1.47 (0.46)	1.48 (0.49)	0.02 (0.07)	0.7 (3.7)	NA
Coordination training								
Exp	Habitual	93	9.0 (1.2)	1.24 (0.11)	1.31 (0.13)	0.08 (0.07)	6.3 (5.2)	0.66
	Fast	133	7.0 (1.4)	1.45 (0.27)	1.56 (0.27)	0.12 (0.14)	8.7 (11.5)	0.73
Con	Habitual	87	9.0 (1.2)	1.22 (0.14)	1.23 (0.13)	0.01 (0.06)	1.3 (4.7)	NA
	Fast	127	7.0 (1.4)	1.43 (0.29)	1.38 (0.25)	–0.05 (0.15)	–4.9 (12.2)	NA
Multimodal training								
Exp	Habitual	340	14.2 (8.0)	1.27 (0.15)	1.32 (0.16)	0.05 (0.07)	4.4 (6.1)	0.77
	Fast	249	12.5 (11.1)	1.43 (0.44)	1.55 (0.46)	0.12 (0.20)	10.5 (15.2)	0.94
Con	Habitual	324	14.2 (8.0)	1.23 (0.16)	1.23 (0.14)	–0.01 (0.04)	–0.2 (3.4)	NA
	Fast	261	12.5 (11.1)	1.39 (0.45)	1.40 (0.45)	0.01 (0.05)	0.8 (3.6)	NA
All								
Exp	Habitual	843	12.4 (12.1)	1.26 (0.16)	1.33 (0.21)	0.07 (0.10)	5.8 (7.5)	0.94
	Fast	750	9.3 (5.3)	1.46 (0.38)	1.58 (0.39)	0.12 (0.14)	9.4 (11.2)	0.89
Con	Habitual	766	12.4 (12.1)	1.22 (0.18)	1.22 (0.17)	0.00 (0.05)	0.1 (4.4)	NA
	Fast	708	9.3 (5.3)	1.43 (0.40)	1.42 (0.45)	–0.01 (0.09)	–1.2 (6.5)	NA

Values other than frequencies and ES are mean (±SD)

*Con* control, *d* distance used to measure gait speed, *ES* between-group effect size (ES ≥0.80 is large), *Exp* experimental, *NA* not applicable because ES is computed between and not within groups, *Pre, m/s* gait speed before the intervention, *Post, m/s* gait speed after the intervention, *SD* standard deviation, *∆, m/s* change in gait speed, *∆,* *%* change in gait speed

Table 3 summarizes the effects of the three types of exercise interventions according to the distance used for the gait test, i.e., short vs. long. Too few studies were available to stratify the data by the three interventions for the long path gait tests. Of 33 studies, 31 used a straight path and 30 of 33 studies used habitual gait speed for the short-distance test. As expected, all of the nine studies used a curved path to test gait speed over a long distance, but the instructions to the subjects were not reported or differed between the studies, e.g., ‘walk as far as possible …’ [100] or ‘walk at a pace similar to which you may use during common daily events’ [111]. Perhaps of all comparisons, interventions improved gait speed the most when it was tested over a long path, by 0.13 m/s or 9.9 % (ES: 1.26). The corresponding values for changes using short-path gait tests were 0.08 m/s, 6.2 %, and an ES of 0.81.

**Table 3:** Effects of exercise interventions on gait speed measured over a short and long distance in healthy old adults

Test	Group	*N*	*d*, m	Pre, m/s	Post, m/s	∆, m/s	∆, %	ES
Short	Exp	1033	14.7 (20.6)	1.31 (0.27)	1.40 (0.35)	0.08 (0.16)	6.2 (10.3)	0.81
Long^a^	Exp	295	479 (138)	1.33 (0.11)	1.46 (0.11)	0.13 (0.10)	9.9 (8.1)	1.26
Short	Con	965	14.7 (20.6)	1.31 (0.30)	1.32 (0.33)	0.01 (0.06)	0.6 (4.1)	NA
Long	Con	281	482 (170)	1.25 (0.21)	1.18 (0.25)	−0.07 (0.12)	−5.5 (9.4)	NA

Values other than frequencies and ES are mean (±SD)

*Con* control, *d* distance used to measure gait speed, *ES* between-group effect size (ES ≥0.80 is large), *Exp* experimental, *Long* gait test using a long distance, *NA* not applicable because ES is computed between and not within groups, *Pre, m/s* gait speed before the intervention, *Post, m/s* gait speed after the intervention, *Short* gait test using a short distance (<30 m), *∆, m/s* change in gait speed, *∆,* *%* change in gait speed

^a^The distance values are not the same for the long tests because the distance covered by subjects in the experimental and control groups differed at baseline for time-dependent measures such as the 6-minute-walk test

We identified ten studies that examined the effects of exercise interventions on the TUG in 304 and 268 healthy old adults in the experimental and control group, respectively. The number of studies was too low to perform an analysis for each of the three interventions. Because the TUG involves standing up from a chair, walking straight, turning 180°, walking straight, and sitting down in a chair, gait speed at baseline, as expected, was slower (0.80 ± 0.20 m/s, *n* = 572) than the gait speed measured over a short but straight-path distance (1.24 ± 0.18, *n* = 1540). The three interventions increased gait speed of 0.82 (0.19) at baseline to 0.92 (0.18) m/s, a gain of 0.10 (0.06) m/s or 13.7 % (8.8) (ES: 0.75) in contrast with the small changes in the control groups (–0.01 m/s ± 0.02; –0.8 % ± 3.4).

Table 4 provides an overall summary of the absolute and relative changes in gait speed and the ESs.

**Table 4:** Summary of the effects of three types of exercise interventions on the gait speed of healthy old adults

	Change in gait speed, m/s				Change in gait speed, %				Effect size			
	RT	CT	MT	All	RT	CT	MT	All	RT	CT	MT	All
All	0.11	0.09	0.09	0.10	9.3	7.6	8.4	8.4	**0.84**	0.76	**0.86**	**0.84**
Hab.	0.09	0.09	0.05	0.07	6.8	6.3	4.4	5.8	1.15	0.66	0.77	**0.94**
Fast	0.12	0.12	0.12	0.12	9.0	8.7	10.5	9.4	**0.90**	0.73	**0.94**	**0.89**
Short				0.08				6.2				**0.81**
Long				0.13				9.9				**1.26**
TUG				0.10				13.7				0.75

*CT* coordination training, *fast* fast gait speed, *Hab* habitual, *habitual* habitual gait speed, *long* distance measured during gait test was long, *MT* multimodal training, *RT* resistance training, *short* distance walked during gait test was short, *TUG* timed-up-and-go test

Effect sizes ≥0.80 are large (indicated in bold)

## Discussion

The main finding of the present systematic review and meta-analysis supported the hypothesis that exercise interventions compared with inactive control can substantially and clinically meaningfully increase the gait speed of even healthy old adults by 0.10 m/s or 8.4 % (ES: 0.84). The primary analysis also confirmed the second somewhat provocative hypothesis that resistance (0.11 m/s or 9.3 %, ES: 0.84), coordination (0.09 m/s or 7.6 %, ES: 0.76), and multimodal training (0.09 m/s or 8.4 %, ES: 0.86) increase gait speed similarly. We discuss these results in the context of functional significance, implications for exercise prescription, and mechanisms of adaptation.

The analyses are based on data from 2495 individuals aged 74 (range 65–83) with typical body mass (69.9 kg), BMI (26.4 m/kg^2^), and without apparent comorbidities per inclusion criteria in the 42 studies (Table 1). Although the 1.22 m/s gait speed observed in the total sample could serve as a reference, we qualify this value by noting that this speed is an aggregate of walking tests administered over short and long distances on a straight or curved path at a habitual and fast pace. Habitual gait speed of 1.24 m/s (*n* = 804) measured at baseline were between the standard values of 1.15 [25] and 1.30 m/s [14] reported previously. The agreement is most likely related to all three studies measuring gait speed over a short and straight course (present study: 12.2 m; Oberg et al. [25]: 5.5 m; Bohannon and Williams [14]: 3–30 m). In contrast, our fast walking speed of 1.44 m/s (*n* = 766) was slower than the standard values of 1.50 and 1.90 m/s because 10 of 29 studies included in our analyses administered the gait test on a curved path, which slows gait. Together, subject and gait speed characteristics of the present sample suggest that the results are relevant to healthy old adults.

Results of the primary analysis confirmed the prediction that the three types of exercise interventions would improve gait speed similarly. This expectation is based on the premise that although healthy old adults present with various sub-clinical neuromuscular and other dysfunctions (see Sect. 1), such impairments and their effects on mobility are evenly distributed among study participants. In the absence of a specific dysfunction, interventions designed to target specific dysfunctions, therefore, exert a general and heterogeneous effect, making it unlikely that any one particular or a multimodal exercise intervention would be superior in increasing gait speed. Qualitatively, this finding agrees with the main conclusion of a previous review, which did not quantify the comparative effects of resistance versus multimodal training through meta-analyses [8]. Indeed, it is possible that our hypothesis, i.e., therapeutic exercise interventions have a similar effect on gait speed, is applicable beyond healthy old adults because study participants of the previous review included patients with chronic health problems, including osteoarthritis, heart disease, peripheral arterial occlusive disease, kidney disease, chronic obstructive pulmonary disease, stroke, fibromyalgia, and obesity [8]. Thus, the emerging idea is that specific exercise interventions (i.e., resistance, coordination, multimodal) will have comparable effects on mobility, at least when measured by gait speed, in analyses that include a population consisting of healthy individuals or a population of patients with diverse dysfunctions because the absence of a specific dysfunction will diminish the specific effects of any one particular exercise training stimulus.

Resistance, coordination, and multimodal interventions increased gait speed by 0.11, 0.09, and 0.09 m/s or 9.3, 7.6, and 8.4 %, respectively (Figs. 3, 4, 5; Tables 1, 4). Compared with a prior exercise review, these changes are substantially greater than the 0.02 and 0.01 m/s increases produced, respectively, by resistance and multimodal training, which were also independent of exercise intensity (high: 0.02 m/s change) and dosage (high: 0.02 m/s) in 32 studies (*n* = 2054) [64]. The overall ES, expressed as a correlation, was *r* = 0.165 (*p* < 0.001) [64], which would correspond to approximately a standardized mean difference of 0.25 (Hedge’s g value), over threefold lower than our overall 0.84 Hedge’s g value (Fig. 1; Table 1). The causes of these large differences in absolute (m/s) and relative (%) values, as well as ES, are unclear. As in the review by Mian et al. [8], Lopopolo et al. [64] also included several studies with patients (hypertension, stroke, balance and strength deficits, post-polio syndrome, heart disease, arthritis, obesity, diabetes, cancer, functional limitations), all of which we excluded. These studies would tend to decrease baseline gait speed and increase the potential for a larger response to the intervention. But this was not the case. While habitual gait speed at baseline, 0.99 m/s, was indeed much lower than our 1.22 m/s, perhaps the main cause of the discrepancy, after re-computing habitual gait speed from table 2 in Lopopolo et al. [64], is the 0.03 m/s change in the control group versus the 0.05 m/s change in the experimental group’s gait, diminishing the ES and net speed improvements caused by the interventions.

The discrepancies between reviews have powerful effects on the interpretation of the data whether or not the improvements in gait speed are clinically meaningful. Given that a change of 0.10 m/s in gait speed is considered substantial relative to self-reported decline in physical function or mobility [134], it is also noted there that 0.05 m/s is a small yet still meaningful change in gait speed. The 0.10 and 0.05 values, recommended as a clinical threshold [134], are far greater than the 0.01 m/s change reported in the review by Lopopolo et al., but these recommended values numerically coincide with the changes observed in the present study. In addition, the hazard ratios and confidence intervals were nearly identical at survival 8 years later for improvements of 0.10 and 0.05 m/s [135]. Because the present review focuses on ‘healthy old adults’ who are walking near or at usual adult gait speed to begin with, the 0.05–0.09 m/s increases in habitual gait speed overall and in response to the three types of interventions represent a functionally important change. This conclusion is well in line with the guideline of 20–30 s for 400-m walk time and 0.03–0.05 m/s for 4-m gait speed and with large changes of 50–60 s for 400-m walk time and 0.08 m/s for 4-m gait speed [136]. The mean age of our study population was 74.2 years, and gait speed loss accelerates from the 1–2 % slowing per decade before the age of 62 years to 12–16 % per decade after the age of 62 years, implying that—if the gait speed outcomes were sustained—exercise interventions could reduce the 0.15 m/s (12 %/decade) in females and 0.21 m/s (16 %/decade) gait speed loss in males by half or more [21]. This analysis assumes that gait speed outcomes are sustained. Based on this assumption, we computed that an intervention-related change of 0.10 m/s may decrease the age-related decline of ~48 % per decade in elderly men and ~66 % per decade in elderly women. While the 0.10 and 0.05 m/s functional cut-off scores seem to gain credence [134, 136, 137], these values must be placed within the context of reliability of gait tests, which are in general high [100, 138–140], but studies also report day-to-day changes of over 43 m, in, for example, the 6-minute walk test that exceed the recommended functional cut-off values [141]. Taken together, the present review found that exercise interventions improve the gait speed of healthy old adults to a degree that is functionally meaningful.

The secondary analyses showed that interventions overall were more effective in improving fast (0.12 m/s, 9.4 %) than habitual gait speed (0.07 m/s, 5.8 %) (Tables 2, 4). These results agree with our tentative hypothesis but are in sharp contrast to the findings of a previous meta-analysis that reported zero intervention effects on fast gait speed [64]. Another review also examined the intervention effects on fast gait but only qualitatively, on a study-by-study basis [8], revealing significant increases in fast gait speed [76, 82–84]. These contradictory findings may in part be due to the variety of fast gait speed instructions [64]. All three training modalities improved habitual gait speed at or above a functionally meaningful level, with resistance (0.09 m/s, 6.8 %) and coordination (0.08 m/s, 6.3 %) training revealing a somewhat higher efficacy than multimodal training (0.05 m/s, 4.4 %). The somewhat lower efficacy of multimodal training provides some cursory evidence for the notion that the effects of the individual elements of multimodal training may not be additive and can perhaps unfavorably interact, diminishing the overall training effect, a phenomenon that has a physiological basis [79]. These results warrant some caution because, in this breakdown analysis, the coordination intervention included only five studies (Fig. 4; Table 4) and in all analyses the gait speed results exhibited low consistencies, illustrated by the poor overlap of the confidence intervals between studies and the significant chi-squared values (Figs. 2, 3, 4, 5).

A remarkably consistent finding was that each of the three interventions improved fast gait speed exactly by 0.12 m/s or about 9 % with medium-large ESs (0.73–0.94) (Tables 2, 4). We interpret these data to mean that (the three types of) exercise interventions are more likely to improve gait speed assessed by a test that imposes a high demand on elements of the neuromuscular system that contribute to gait speed generation. This interpretation is consistent with another result of the secondary analysis showing that gait test administered over a long path, presumably also demanding for many old adults, produced the single largest ES (1.26) and absolute change (0.13 m/s) (Tables 2, 4). In contrast, TUG revealed one of the lowest ESs (0.75) of the 15 comparisons, with an average 0.10 m/s change.

The results of this review have some implications for exercise prescription. It seems that healthy old adults and care providers could select among these exercise programs freely but certainly dictated by individual preferences, experience, social context, and medical precaution. As stated throughout this paper, many if not most old adults who are categorized as healthy present with various sub-clinical medical and health problems, among them emerging mobility dysfunction, dynapenia, sarcopenia, obesity, arthritis, diabetes, and would strongly benefit from an exercise program tailored to individual needs [142–146]. Still, the review provides a conceptual basis that resistance, coordination, and a multimodal training program would most likely afford some clinically meaningful benefits in terms of walking speed for most if not all healthy old adults and help slow the loss of gait speed or delay its onset.

### Limitations and Recommendations

The present review cannot address perhaps the most intriguing question concerning the physiological and biomechanical mechanisms of how the newly acquired physical abilities actually convert into higher gait speed [12]. The results seem to suggest that resistance and coordination training programs, taking just the two most dissimilar exercise interventions, are similarly effective but probably act through different mechanisms that underlie gait speed increases. In a recent study, light-load high-velocity leg-press training modified the contribution of five muscle groups to gait speed so that hip extensors and ankle plantar flexors were the only significant predictors of habitual and fast gait speed, respectively [129]. Considering this latter result and the robust observation of a preferential reduction in ankle function measured during gait in old adults [27, 147–151], with a few exceptions [107], the vast majority of resistance training studies target the knee extensors (for a review, see Raymond et al. [152]). While the timing and coordination approach to gait speed improvement has a solid conceptual basis and capitalizes on a long history of treating old adults’ mobility disability [16, 28, 106], experimental evidence is lacking to support any particular mechanism mediating increases in gait speed after such interventions [12]. Considering the massive ongoing efforts to combat mobility disability in the rapidly increasing number of old adults worldwide [41, 67], there is an urgent need to extend the current sporadic evidence [107, 153–155] and perform biomechanical and neurophysiological studies that examine the changes in joint torques and powers, muscle activation patterns, synergies, and other mechanistic indices measured during gait to better understand how exercise interventions change gait behavior [12].

Given only one study met our criteria for the review, we were unable to determine the effects of leg power training on gait speed even though recent studies strongly promote this form of intervention for the re-training of the aged neuromuscular system and mobility [68, 72, 147, 156–158]. Therefore, there is a need to update the findings of the present review when the number of power interventions is sufficiently high to arrive at a state-of-the-art statement. The low study numbers also limited the scope of the review because we were unable to stratify the effects of the three intervention types for distance walked and the TUG. Unlike previous studies, we did not examine whether the responses to the three interventions scaled according to a dose-response relationship. We also note the limitation that even though most studies did report the sex breakdown in the subject characteristics section, virtually none of the studies reported gait speed by sex. Therefore, it is not entirely clear whether or not the sex distribution (421 males, 695 females, bottom row, Table 1) biases the conclusions. Future studies and reviews should also address a conceptual limitation of the present review. Because we examined healthy old adults, it was not possible to address a cardinal issue whether the herein reviewed intervention-induced gait speed increases would actually reduce mobility disability later in life in currently healthy old adults.

## Conclusions

This systematic review and meta-analysis tested the hypothesis that commonly used exercise interventions can functionally meaningfully increase healthy old adults’ gait speed and that the training effects produced by resistance, coordination, and multimodal interventions with respect to gait speed are similar. Based on data from 42 studies, the overall increase in gait speed was 0.10 m/s or 8.4 % with a large ES of 0.84 in 2495 healthy old adults aged 74.2 years. Additional analyses revealed that resistance (0.09 m/s, 6.8 %) and coordination training (0.08 m/s, 6.3 %) were somewhat more effective than multimodal training (0.05 m/s, 4.4 %) to increase habitual gait speed, but all three modalities increased fast gait speed dramatically and numerically identically by 0.12 m/s. The single highest intervention effects occurred when gait speed was tested on a long path: 0.13 m/s, 9.9 % (ES of 1.26). Commonly used exercise interventions can increase the habitual and fast gait speed of healthy old adults in substantial and clinically meaningful ways.

## Electronic supplementary material

Supplementary material 1 (DOCX 145 kb)

Supplementary material 2 (DOCX 91 kb)
