# Supplementary material for: Effects of Three Types of Exercise Interventions on Healthy Old Adults’ Gait Speed: A Systematic Review and Meta-Analysis
Source: Sports Med. 2015 Aug 19;45:1627–43. doi: 10.1007/s40279-015-0371-2 (PMC4656792; doi:10.1007/s40279-015-0371-2)
Supplement: Supplementary file 1 — Supplementary material 1 (DOCX 145 kb) [file 40279_2015_371_MOESM1_ESM.docx]

Electronic Supplementary Material Appendix S1. The PubMed search syntax used in the present review to identify resistance, power, coordination, and multimodal exercise intervention designed to improve healthy old adults’ habitual and fast gait speed. This search syntax was adapted to Web of Knowledge and Cochrane databases (not shown).

(“Resistance training" OR "Strength training" OR "Weight-bearing exercise program" OR "Power training" OR "Coordination training" OR "Balance training" OR "Proprioceptive training" OR "Sensorimotor training" OR "Instability training" OR "Perturbation training" OR "Tai Chi" OR "Dance training" OR "Gait training" OR "Gait retraining" OR "Functional training" OR "Neuromuscular training" OR "Combination training" OR "Multifactorial training" OR "Multimodal training" OR "Endurance training" OR "Aerobic training" OR "Endurance exercise training" OR "Aerobic exercise training" OR "Gait intervention" OR "Gait exercise" OR "Endurance walking training" OR "Endurance gait training" OR "Physical endurance training") AND (Gait OR Walking) NOT Patient NOT Disease NOT Stroke NOT Diabetes NOT Neuropathy NOT Amputation NOT "Multiple sclerosis" NOT Cerebral palsy" NOT Parkinson NOT Cancer NOT Obese NOT Osteoarthritis NOT Fracture NOT Dysfunction NOT “Cognitively impaired” NOT Frail NOT Demented NOT "Pilot study"

Pubmed filters:

- Randomized controlled trial
- Full text
- 1984-2014
- Aged 65+
- English
