# Supplementary material for: Effects of Three Types of Exercise Interventions on Healthy Old Adults’ Gait Speed: A Systematic Review and Meta-Analysis
Source: Sports Med. 2015 Aug 19;45:1627–43. doi: 10.1007/s40279-015-0371-2 (PMC4656792; doi:10.1007/s40279-015-0371-2)
Supplement: Supplementary file 2 — Supplementary material 2 (DOCX 91 kb) [file 40279_2015_371_MOESM2_ESM.docx]

Electronic Supplementary Material Appendix S2. Physiotherapy evidence database (PEDro) scores of the reviewed intervention studies.

Author Reference a b c d e f g h j j k l

Baker 2007 [94] 1 1 1 1 0 0 1 1 1 1 1 8

Barret 2002 [95] 1 1 1 1 0 0 1 1 1 1 1 8

Brandon 2000 [96] 1 1 0 1 0 0 0 1 0 1 1 5

Brandon 2004 [97] 1 1 0 1 0 0 0 1 0 1 1 5

Bunout 2001 [98] 1 1 0 1 0 0 0 0 0 1 1 4

Capodaglio 2007 [99] 1 0 0 0 0 0 0 0 0 1 1 2

Cavani 2002 [100] 0 0 0 1 0 0 0 1 0 1 1 3

Cress 1999 [101] 1 1 0 1 0 0 0 0 0 1 1 4

De Vreede 2005 [89] 1 1 0 1 0 0 1 1 0 1 1 6

Englund 2005 [102] 1 1 0 1 0 0 0 1 0 1 1 5

Fatouros 2005 [103] 1 1 0 1 0 0 0 1 0 1 1 5

Granacher 2010 [105] 1 1 0 1 0 0 0 1 0 1 1 5

Granacher 2012 [106] 1 1 0 1 0 0 0 1 0 1 1 5

Granacher 2013 [104] 1 1 0 1 0 0 0 1 1 1 1 6

Hartmann 2009 [107] 1 1 0 1 0 0 0 0 1 1 1 5

Hartmann 2010 [108] 1 1 0 1 0 0 0 1 1 1 1 6

Henwood 2005 110] 1 0 0 1 0 0 0 1 0 1 1 4

Henwood 2006 [111] 1 1 0 1 0 0 0 1 0 1 1 5

Henwood 2008 [109] 1 1 0 1 0 0 0 0 0 1 1 4

Judge 1993 [112] 1 1 0 1 0 0 0 1 0 0 1 4

Judge 1994 [113] 1 1 0 1 0 0 1 1 1 1 1 7

Kalapotharakos 2005 [114] 1 1 0 1 0 0 1 1 0 1 1 6

Kamide 2009 [115] 1 1 0 1 0 0 1 1 0 1 1 6

Lamoureux 2003 [116] 1 1 0 1 0 0 0 1 0 1 1 5

Lamoureux 2003 [117] 1 1 0 1 0 0 0 1 0 1 1 5

Lord 1996 [118] 0 1 0 1 0 0 0 0 0 1 1 4

Magistro 2014 [119] 1 1 0 1 0 0 0 1 0 1 1 5

Malatesta 2010 [22] 1 0 0 1 0 0 0 1 0 1 1 4

Nelson 2004 [120] 1 1 0 1 0 0 1 1 0 1 1 6

Nichols 1995 [121] 0 1 0 0 0 0 0 1 0 1 1 4

Okumiya 1996 [122] 1 1 0 1 0 0 1 1 0 1 1 6

Puggaard 2003 [123] 1 0 0 1 0 0 0 0 0 1 1 3

Ramsbottom 2004 [124] 1 1 0 1 0 0 0 1 1 1 1 6

Schlicht 2001 [83] 1 1 0 1 0 0 0 1 0 1 1 5

Sipilä 1996 [76] 1 1 0 1 0 0 0 0 0 1 1 4

Skelton 1995 [125] 1 1 0 1 0 0 0 1 0 1 1 5

Topp 1993 [126] 0 1 0 1 0 0 0 1 0 1 1 5

Topp 1996 [127] 1 1 0 0 0 0 0 0 0 1 1 3

Toraman 2004 [128] 0 1 0 1 0 0 0 1 0 1 1 5

Uematsu 2014 [129] 0 1 0 1 0 0 0 1 0 1 1 5

Wolfson 1996 [130] 1 1 0 1 0 0 1 1 0 1 1 6

Zhuang 2014 [131] 1 1 1 1 0 0 0 1 0 1 1 6

Mean 5.0

SD 1.2

a, Eligibility criteria specified

b, Random allocation

c, Allocation concealed

d, Groups similar at baseline

e, Blinding of subjects

f, Blinding of therapists

g, Blinding of aassessors

h, Dropout < 15%

i, Intention0to0treat method

j, Statistical between0group comparisons

k, Point measures and measures of variability

l, PEDro score
